# The role of Espin in the stereocilia regeneration and protection in Atoh1‐overexpressed cochlear epithelium

**DOI:** 10.1111/cpr.13483

**Published:** 2023-04-21

**Authors:** Xuechun Yang, Jieyu Qi, Liyan Zhang, Fangzhi Tan, Hongming Huang, Chunlai Xu, Yong Cui, Renjie Chai, Peina Wu

**Affiliations:** ^1^ School of Medicine, South China University of Technology Guangzhou China; ^2^ Department of Otolaryngology Guangdong Provincial People's Hospital (Guangdong Academy of Medical Sciences), Southern Medical University Guangzhou China; ^3^ State Key Laboratory of Bioelectronics, Department of Otolaryngology Head and Neck Surgery Zhongda Hospital, School of Life Sciences and Technology, Advanced Institute for Life and Health, Jiangsu Province High‐Tech Key Laboratory for Bio‐Medical Research, Southeast University Nanjing China; ^4^ Department of Otolaryngology Heyuan City People's Hospital, Jinan University Guangzhou China; ^5^ Co‐Innovation Center of Neuroregeneration, Nantong University Nantong China; ^6^ Department of Otolaryngology Head and Neck Surgery Sichuan Provincial People's Hospital, University of Electronic Science and Technology of China Chengdu China; ^7^ Institute for Stem Cell and Regeneration Chinese Academy of Science Beijing China; ^8^ Beijing Key Laboratory of Neural Regeneration and Repair Capital Medical University Beijing China

## Abstract

Hair cells (HCs) in mammals cannot spontaneously regenerate after damage. Atoh1 overexpression can promote HC regeneration in the postnatal cochlea, but the regenerated HCs do not possess the structural and functional characteristics of HCs in situ. The stereocilia on the apical surface of HCs are the first‐level structure for sound conduction, and regeneration of functional stereocilia is the key basis for the reproduction of functional HCs. Espin, as an actin bundling protein, plays an important role in the development and structural maintenance of the stereocilia. Here, we found that the upregulation of Espin by AAV‐ie was able to induced the aggregation of actin fibres in Atoh1‐induced HCs in both cochlear organoids and explants. In addition, we found that persistent Atoh1 overexpression resulted in impaired stereocilia in both endogenous and newly formed HCs. In contrast, the forced expression of Espin in endogenous and regenerative HCs was able to eliminate the stereocilia damage caused by persistent Atoh1 overexpression. Our study shows that the enhanced expression of Espin can optimize the developmental process of stereocilia in Atoh1‐induced HCs and can attenuate the damage to native HCs induced by Atoh1 overexpression. These results suggest an effective method to induce the maturation of stereocilia in regenerative HCs and pave the way for functional HC regeneration via supporting cell transdifferentiation.

## Graphical abstract

Persistent Atoh1 overexpression results in impaired stereocilia in both endogenous and regenerative hair cells (HCs). The enhanced expression of Espin optimizes the developmental process of stereocilia in Atoh1‐induced HCs. The upregulation of Espin attenuates the damage to stereocilia caused by Atoh1 overexpression.

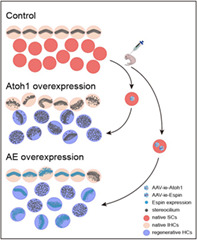

## 1 INTRODUCTION

Hair cell (HC) damage is one of the main causes of sensorineural hearing loss, and hearing loss is irreversible in mammals because adult HCs cannot regenerate spontaneously.^1^ In contrast, non‐mammalian vertebrates can spontaneously restore auditory and vestibular HCs by triggering the regeneration of adjacent supporting cells (SCs).^2^ HCs are the sensory cells responsible for sound perception in vertebrates, and auditory HCs include one row of inner hair cells (IHCs) and three rows of outer hair cells (OHCs). The HCs that detect sound have a bundle of hair‐like protrusions called stereocilia at their top. The stereocilia are a typically arranged in a step‐like structure formed by the arrangement of parallel actin bundles, and these can transform mechanical stimulation into electrical activity and thus are indispensable for HCs to perform their normal physiological functions.^3^ This process, known as mechanoelectric transduction, is critical to hearing, and thus dysplasia or damage to the stereocilia can seriously affect the function of HCs, and vertebrates become deaf when a large number of HCs are dysfunctional.

Because OHCs, IHCs and SCs originate from the same progenitors during inner ear development, SCs are considered to be a promising resource for mammalian HC regeneration,^4^ and SCs have now been shown to regenerate HCs under certain conditions.^5^, ^6^ IHCs and OHCs are believed to originate from a common pool of Atoh1+ progenitors.^7^, ^8^ Atho1 (Math1) is a helix‐ring‐helix transcription factor and is a key regulator of cochlear HC development and regeneration. Deletion of the *Atoh1* gene leads to the failure of HC formation, while its overexpression in SCs from neonatal mice causes ectopic HC production.^9^ Therefore, Atoh1 is considered to be a key transcription factor for HC formation, and its overexpression leads to the efficient conversion of SCs to HCs. However, Atoh1 overexpression alone is not sufficient to generate the morphological features of mature HCs, such as stereocilia.^10^, ^11^ HCs without mature stereocilia are nonfunctional, so these HCs induced by Atoh1 are unable to undergo mechanoelectric transduction of sound like native HCs. Thus our goal is to find ways to normalize the stereocilia morphology and function of regenerative HCs.

Stereocilia contain three classes of actin‐bundling proteins—espins, fascins and fimbrin/plastins—with the espin family being involved in stereociliary morphologenesis.^12^ Espins bind to and cross‐link actin filaments into parallel actin bundles in vitro with high affinity and are unaffected by calcium concentrations,^2^, ^13^, ^14^ and this activity induces intracellular parallel actin bundles to lengthen in a concentration‐dependent manner.^15^, ^16^ The Jerker mouse model shows that stereocilia degenerate in the absence of Espin, resulting in HC dysfunction and deafness,^17^, ^18^ and it has been shown that Espin is important for the growth and maintenance of the actin‐based protrusions of inner ear neuroepithelial cells.^19^ Researchers used espin‐BAC transgenic mice to overexpress Espin in the cochlea. Until P60, it was found that the stereocilia of HCs became thicker and the length of different rows of stereocilia increased differently, but no hearing function was tested.^20^ Furthermore, an in vitro study found that Espin overexpression in regenerated vestibular HCs induced by Notch inhibition led to an increase in hair‐bundle‐like structures, suggesting that Espin overexpression might be involved in the injury or regeneration of stereocilia.^21^ However, only in vitro experiments were conducted, and the effect on stereocilia in IHCs and OHCs was not studied in this study. It is important to note because stereocilia in the HCs of the organ of Corti are somewhat different from those in vestibular HCs.

Here, we successfully delivered Atoh1 to the mouse inner ear and induced HC regeneration using a novel AAV‐ie that targets inner ear HC precursor cells, the SCs, while at the same time inducing overexpression of exogenous Espin in the SCs. Continuous overexpression of Atoh1 in the organ of Corti not only promoted HC regeneration, but also damaged the stereocilia of native and regenerative HCs. We investigated the effects of Espin on the stereocilia of both in situ and Atoh1‐induced HCs in the organ of Corti in vivo. A stepped structure of stereocilia was observed in the regenerative HCs, and we found that the presence of Espin attenuated Atoh1‐induced damage to stereocilia of in situ HCs. Our study thus shows that enhanced expression of Espin optimizes the development of stereocilia in Atoh1‐induced HCs and prevents the degeneration of stereocilia in endogenous HCs that results from Atoh1 persistent overexpression.

## 2 MATERIALS AND METHODS

### 2.1 Animals

Wild‐type neonatal (postnatal day (P)1) mice in the FVB/N background were used. All experiments were approved by the Institutional Animal Care and Use Committee of Southeast University, China.

### 2.2 Organoid culture

P1 FVB mice were sacrificed and the temporal bones were dissected and placed in pre‐cooled 1× HBSS. We dissected the cochleae under a stereo microscope, removed the basilar membranes, and placed them in a 1.5 mL Eppendorf tube containing pre‐cooled 1× HBSS. The basilar membranes were washed three times with PBS. A total of 100 μL pre‐warmed 0.25% trypsin (final concentration of 0.125%) was added and incubated for 7 min in a 37°C incubator. The digestion was stopped by addition of 100 μL trypsin inhibitor at a final concentration of 2.5 mg/mL. The basilar membranes were dispersed into a single‐cell suspension by pipetting with 200 μL blunt tips, and the cells were filtered through a 40 μm cell strainer. The filtrate was centrifuged (1000 × *g* for 7 min), and the supernatant was discarded to obtain the cell pellet. The cells were resuspended in culture medium and then mixed in Matrigel (at a concentration of 33%) (Corning, USA). The resuspended cell suspension was seeded on a 10 mm slide in a 24‐well plate, and the plate was placed into an incubator for 30 min and culture medium was added after the Matrigel solidified. First, we cultured the cells to proliferate for 8 days, and the medium was DMEM/F12, N2, B27, EGF, FGF, IGF, VPA and Ampicillin. On the ninth day, we changed to another culture medium, which was DMEM/F‐12, N2, B27, EGF, FGF, IGF, CHIR, LY and Ampicillin. At the same time, AAV was added to the culture medium for 48 h. In the AAV‐At group only AAV‐Atoh1‐mNeonGreen (2 × 10^10^ GC/well) was added, while in the AAV‐AE group both AAV‐Atoh1‐mNeonGreen (2 × 10^10^ GC/well) and AAV‐Espin‐HA (2 × 10^10^ GC/well) were added. The cultures were incubated for another 11 days before sample collection.

### 2.3 Explant culture

The P1 mice were euthanized and decapitated, and their heads were placed in 75% ethanol and quickly transferred to pre‐cooled HBSS. The temporal bones were dissected out, the cochlea was isolated from the temporal bones using sterile procedures in ice‐cold HBSS, and the basilar membranes were removed with tweezers. Explants of the organ of Corti were placed intact on polyphenol‐protein‐coated cover glasses (Cell‐Tak Cell and Tissue Adhesive, Corning, USA) and maintained in four‐well culture dishes (Corning, USA) in culture medium composed of DMEM/F‐12 supplemented with N2, B27, and Ampicillin. The explant tissues were incubated at 37°C in an atmosphere at 5% CO_2_. After 12 h of incubation, AAV was added to each well and incubated for 48 h, and then the culture medium was replaced with fresh culture medium.

### 2.4 Inner ear round window membrane injection

The P1 mice were anaesthetised by hypothermia, and their body temperature was temporarily extremely reduced. After determining that the mouse was successfully anaesthetised, a post‐auricular incision of about 4–5 mm was made. The submandibular gland was gently lifted to expose the neck muscles underneath, and the muscles were separated layer by layer to find where the facial nerve passed by the auditory bulla. After identifying the round window membrane, a glass micropipette containing AAV was slowly inserted into the cochlea through the membrane. Mice in the AAV‐At group were injected only with AAV‐Atoh1‐mNeonGreen (1.5 × 10^10^ GC/ear), while both AAV‐Atoh1‐mNeonGreen (1.5 × 10^10^ GC/ear) and AAV‐Espin‐HA (7.8 × 10^9^ GC/ear) were injected into the mice of the AAV‐AE group. The mice in the control group were wild type without AAV injected. After successful injection, the incision was sealed with tissue adhesive (3M Vetbond), and the animals were placed in a plastic box in a pre‐heated water bath to recover from hypothermia. The total operation time for each animal was 5–6 min.

### 2.5 Immunofluorescence (IF)

The cochleae were harvested and fixed for 30 min at room temperature with 4% paraformaldehyde in 0.1 M phosphate buffer (PBS). After rinsing with PBS, the cochleae were decalcified with 0.5 mM EDTA for 6 h. Under a microscope, the cochleae were dissected and the basilar membrane was removed and placed on a glass slide for subsequent staining.

After being permeabilized in 1% Triton X‐100 in PBS (PBST) for 30 min at room temperature, the samples were blocked with 10% donkey serum in 0.5% PBST for 1 h. The HCs were labelled with the primary antibodies at 4°C overnight, including rabbit antibody against Myo7a (1:1000 dilution; Abcam) and rat antibody against HA (1:100 dilution; R&D). After being washed with PBS to remove the unbound primary antibodies, the samples were incubated for 1 h at room temperature with a donkey anti‐rabbit secondary antibody (1:400 dilution; Invitrogen), an anti‐phalloidin antibody (1:1000 dilution; Thermo Fisher), and 4′,6‐diamidino‐2‐phenylindole dihydrochloride (DAPI), all of which had been diluted in PBST, to visualize Myo7a, phalloidin, and cell nuclei, respectively. Finally, the samples were washed three times with PBS and sealed with DAKO fluorescence mounting medium.

### 2.6 Scanning electron microscopy (SEM)

Cochlear specimens from P14 mice were fixed in fresh 2.5% glutaraldehyde (in PBS, pH 7.2) at 4°C overnight and decalcified in 0.5 mM EDTA (pH 8.0) for 4 h. The cochlear basilar membrane was cut into pieces and post‐fixed in 1% osmic acid (Ted Pella, Inc) for 2 h at room temperature. Specimens were treated with 2% tannic acid before dehydration in an ethanol gradient (30%, 50%, 70%, 80%, 90% and 100%) and critical‐point drying with liquid CO2 (CPD300, Leica). After platinum‐coating in an electrically conductive carbon tape, the cochlear tissues were observed in random fields using a field‐emission scanning electron microscope (SEM, NNS450, FEI).

### 2.7 Real‐time qPCR

The cochlear tissues were ground, and RNA was extracted with an RNA Mini Kit (Thermo Fisher). cDNA was created with a RevertAid First Strand cDNA Synthesis Kit (Thermo Fisher), and qPCR was performed using ChamQ SYBR qPCR Master Mix (Vazyme). The gene primers are shown in Table 1.

**TABLE 1:** Primers used for quantitative real‐time PCR.

Gene	Forward primer (5′‐3′)	Reverse primer (5′‐3′)
*Atoh1*	GAGTGGGCTGAGGTAAAAGAGT	GGTCGGTGCTATCCAGGAG
*Espin*	GCCTGGAGACGAGACAT	AAGATGACCTGTCGCTG
*Fscn2*	CTGTGAGGCAAGGAATCAATGT	CCCCAGTGCTGGAATAGAAAGT
*Pls1*	AGGAGAGGACATTCCGCAACT	GGTACGGAGGCTTGTTCACC
*Fscn1*	GACTGCGAAGGTCGCTACC	CTGATCGGTCTCTTCATCCTGA
*Twf2*	TCATCATTGAGGACGAACAGC	CTCTTGGGCGTCTAGCAGT
*Actn4*	ATGGTGGACTACCACGCAG	CAGCCTTCCGAAGATGAGAGT
*Tpm1*	CAGAAGGCAAATGTGCCGAG	TCCAGCATCTGGTGCATACTA
*Gsn*	ATGGCTCCGTACCGCTCTT	GCCTCAGACACCCGACTTT
*Pfn2*	CTACGTGGATAACCTGATGTGC	TCGCAGTAGCCGACAATGG
*Fzd3*	ATGGCTGTGAGCTGGATTGTC	GGCACATCCTCAAGGTTATAGGT
*Vangl2*	ACTCGGGCTATTCCTACAAGT	TGATTTATCTCCACGACTCCCAT
*Dvl3*	GTCACCTTGGCGGACTTTAAG	AAGCAGGGTAGCTTGGCATTG
*Ptk7*	CCAAGCCGCTATCGTCTTTAT	GACAGGCACCCCATTGAGC
*Gpsm2*	ACCATTCCTTTCATGTCCGCT	GGCAGTCCCCTGATTTACATAGA
*Six1*	ATGCTGCCGTCGTTTGGTT	CCTTGAGCACGCTCTCGTT
*Dvl2*	GGTGTAGGCGAGACGAAGG	GCTGCAAAACGCTCTTGAAATC
*Ccdc88c*	CAGGAGGTGACCCACAACC	AGTCTGCGGAGGTGGAACA

### 2.8 Statistics

All data were analysed with GraphPad Prism 9 using two‐tailed, unpaired Student's *t*‐tests when comparing two groups. All data were expressed as either a percentage or as the mean ± SD. A *p*‐value <0.5 was considered statistically significant.

## 3 RESULTS

### 3.1 Espin promotes the growth of cilia in regenerated HCs in cochlear organoid and explant culture

**FIGURE 1:**
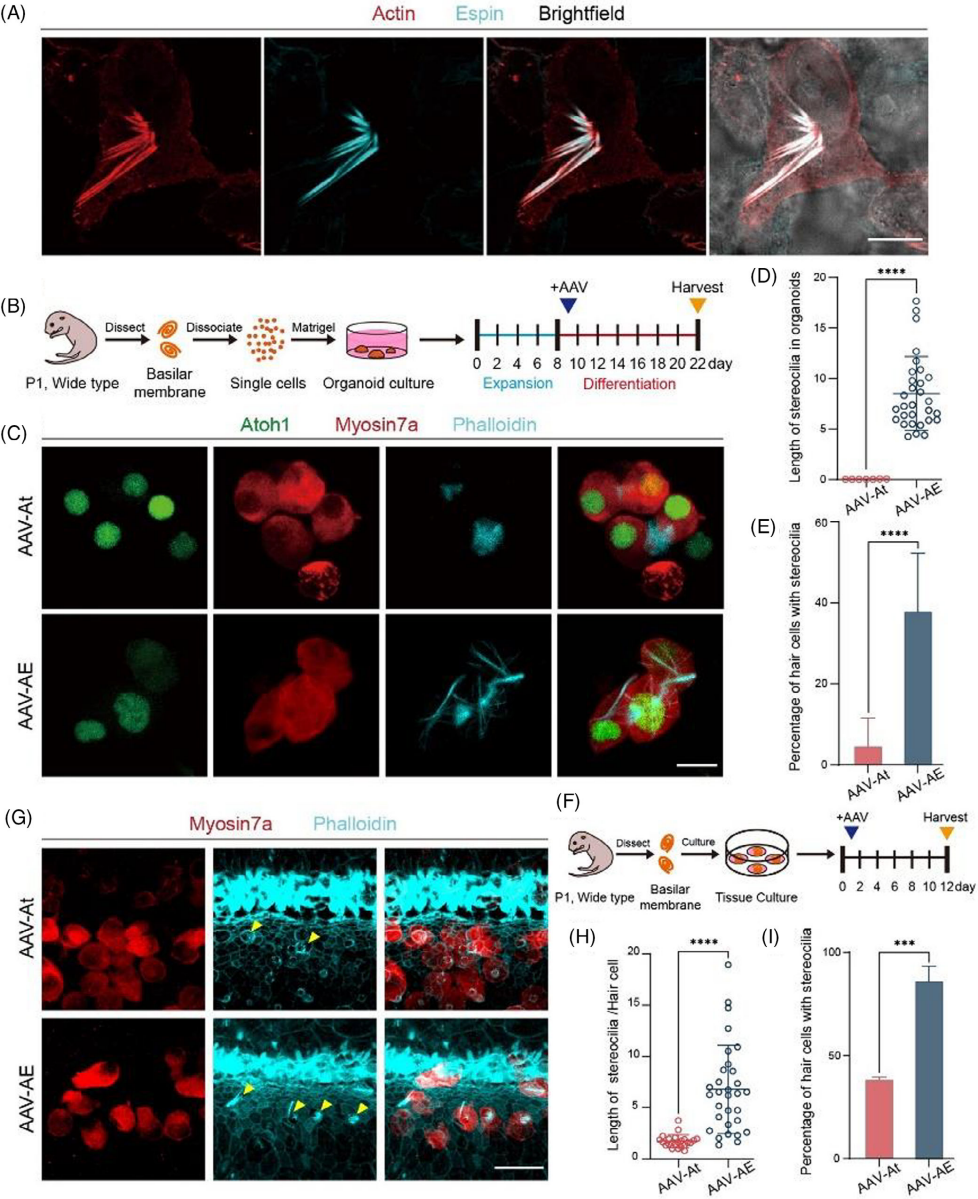
Overexpression of Espin in vitro. (A) Confocal images of Espin‐HA (cyan), actin (red) and brightfield in HEK293T cells. Scale bar, 10 μm. (B) Schematic for the culturing cochlear organoids. (C) Representative confocal images of Atoh1‐mNeonGreen (green), myosin7a (red) and F‐actin (cyan) in cultured organoids. Scale bar, 10 μm. (D) Stereocilia length of hair cells was measured by ImageJ. (E) The proportion of hair cells with stereocilia in organoids was calculated. (F) Schematic of explant culture. (G) Representative confocal images of myosin7a (red) and F‐actin (cyan) in cultured basilar membranes. Scale bar, 20 μm. (H) The stereocilia length of neonatal hair cells was measured. (I) The proportion of regenerated hair cells with stereocilia in explants was calculated. ***p < 0.001; ****p < 0.0001.

To explore the effects of Espin on the stereocilia of regenerated HCs in vitro, we first validated it in HEK293T cells. The immunofluorescence staining results showed that only HEK293T cells with Espin overexpression grew cilia, while adjacent HEK293T cells that were not transduced had no cilia (Figure 1A). This indicated that Espin plays a role in promoting actin formation.

We then used Matrigel to construct a three‐dimensional in vitro environment to explore the effects of Espin upregulation on HCs in organoids.^22^ The literature suggests that some of the SCs, as HC precursor cells, are able to differentiate into HCs in response to upregulated Atoh1 expression,^10^, ^23^ so we overexpressed Atoh1 to promote precursor cell differentiation in organoids. The AAV‐At group showed no cilia structure on the HCs, while the cilia of HCs in the AAV‐AE group elongated significantly but did not form a similar stereocilia structure as they did in vivo (Figure 1C). Compared with AAV‐At group, the cilia of HCs in the AAV‐AE group were significantly longer and the proportion of HCs growing cilia in organoids was much higher (Figure 1D,E). These results indicated that overexpressing Espin promoted the formation and growth of cilia on HCs.

Next, in vitro cochlear basilar membrane culture was performed. AAV was added in the same amounts as for the organoid cultures, and we observed the stereocilia of HCs regenerated by overexpression of Atoh1 (Figure 1F). As indicated by the arrows in the figure, the stereocilia of regenerated HCs in the AAV‐At group were short, while the stereocilia in the AAV‐AE group were clustered and were more similar to the morphology of stereocilia in situ (Figure 1G). The statistical analysis showed that compared to the AAV‐At group the stereocilia of the regenerated HCs in the AAV‐AE group were not only longer, but there was also a higher proportion of regenerated HCs with stereocilia (Figure 1H,I). These results showed that Espin could enable the formation and growth of stereocilia in regenerated HCs induced by Atoh1 overexpression in vitro.

### 3.2 Espin promotes the morphological development of stereocilia in Atoh1‐induced HCs

Given that Espin promotes cilia growth in regenerated HCs in vitro, we next performed in vivo experiments. In recent years, there have been many studies seeking to promote the number and functional maturity of regenerated HCs by combining transcription factors such as Gata3, Pou4f3, p27Kip1, lsl1 and Gfi1/Pou4f3 with Atoh1. These studies have shown that Pou4f3 and Gfi1 can enhance the regenerative function of Atoh1 and can promote the relative physiological maturation of neonatal HCs.^24^, ^25^ Thus, we wanted to explore what effect other genes might have when combined with Atoh1. As reported in a previous study, Espin plays a key role in the development of stereocilia,^19^ and thus when Espin is overexpressed in vivo there is no doubt that neonatal HCs will be affected.

**FIGURE 2:**
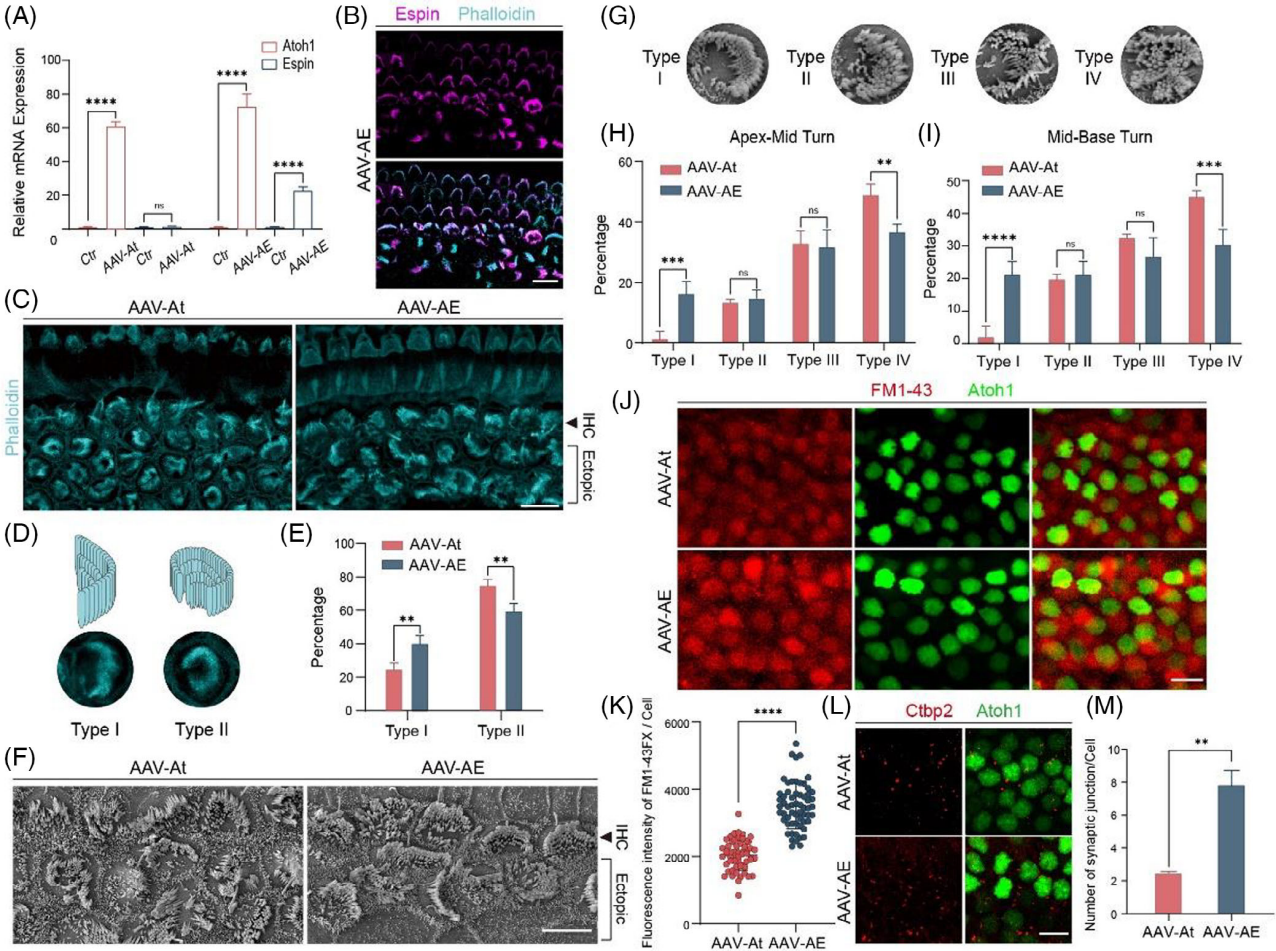
The morphology and function of stereocilia in regenerated hair cells. (A) Real‐time qPCR detected the expression of Espin and Atoh1 carried by AAV‐ie in vivo. (B) Confocal images of Espin‐HA (magenta) and F‐actin (cyan) in P7 mice injected with AAV‐Atoh1 and AAV‐Espin. Scale bar, 10 μm. (C) Representative confocal images of F‐actin (cyan) in P14 mice injected with AAV‐Atoh1 alone or with both AAV‐Atoh1 and AAV‐Espin. Scale bar, 10 μm. (D) Schematic and images of regenerated hair cell typing based on stereocilia morphology. (E) Typing was based on stereocilia immunofluorescence results in the regenerated hair cells. (F) Representative SEM images of neonatal hair cells. Scale bar, 5 μm. (G) Representative diagram of stereocilia typing. (H) Proportions of stereocilia morphology types of regenerated hair cells in the apex to middle of the cochlea. (I) Proportions of stereocilia morphology types of regenerated hair cells in the middle to base of the cochlea. (J) Regenerated hair cells of P14 cochleae in two groups took up FM1‐43FX (red). Scale bar, 10 μm. (K) The fluorescence intensity of FM1‐43FX uptake analysis. (L) Representative confocal images of Atoh1‐mNeonGreen (green) and Ctbp2 (red) in P21 mice injected with AAV. Scale bar, 10 μm. (M) The number of synapses was counted by ImageJ. ns, no significance. **p < 0.01; ***p < 0.001; ****p < 0.0001.

Due to the complexity of the in vivo environment, we need to ensure that the virus is successfully overexpressed after being injected into the cochlea in order to study the effects of virus‐delivered genes on neonatal HCs. The P7 cochleae were collected from all groups, and RNA was extracted and reverse‐transcribed to cDNA to verify the relative expression levels of the genes. QPCR results showed that expression of Atoh1 in the two groups was 60–80 times greater than the control group, and the expression of Espin in the AAV‐AE was about 20 times that of the control and AAV‐At groups (Figure 2A). The basilar membrane was dissected for immunofluorescence staining, and the results showed that Espin was expressed in most stereocilia of neonatal HCs and was also expressed in most stereocilia of IHCs and OHCs in situ (Figure 2B).

Because the stereocilia of regenerated HCs at P7 were immature and quite different from those of HCs in situ (Figure 2B), we dissected the cochlea at P14 to observe the stereocilia of neonatal HCs. Phalloidin staining showed a significant difference in the morphological development of stereocilia in the regenerated HCs between the two groups (Figure 2C). According to the degree of difference in morphology compared to in situ HCs, the stereocilia of the regenerated HCs were divided into two types, with type I being arc‐shaped and type II being ‘C’‐shaped (Figure 2D). The proportion of type I stereocilia in the AAV‐AE group was significantly higher than that in the AAV‐At group, and the proportion of type II stereocilia was lower than that in the AAV‐At group (Figure 2E). The morphology of type I stereocilia was closer to that of in situ IHCs, indicating that the stereocilia development of neonatal HCs in the AAV‐AE group was closer to that of in situ HCs.

To get a clearer picture of the morphology of HC stereocilia, we visualized the stereocilia by SEM (Figure 2F). Compared to IF, the morphology of stereocilia shown by SEM was very clear, so we had more precise classification of stereocilia bundle. Type I were neatly arranged like arc‐shape, and the overall morphology was similar to that of in situ inner HCs. Type II were neatly arranged, but had misarranged bundles on the open side of arc‐shape. Type III were only slightly arranged and were out of order, but the overall shape was similar to ‘C’. Type IV were disarrayed and had no clear morphology (Figure 2G). The proportion of type I in the AAV‐AE group was significantly higher than that in the AAV‐At group, while the proportion of type IV was significantly reduced in the AAV‐AE group, and there was no significant difference in the proportions of type II and III between the two groups (Figure 2H,I). Type I stereocilia morphology was closer to in situ IHCs, and type IV was very different, indicating that regenerated HCs in the AAV‐AE group had a higher degree of stereocilia development than regenerated HCs in the AAV‐At group. Together, these results suggest that Espin can significantly improve the stereocilia structure of Atoh1‐induced HCs.

In terms of the function of regenerative HCs' stereocilia, we supplemented the FM1‐43 uptake experiment of regenerated HCs at P14 (Figure 2J). By comparing the fluorescence intensity of FM1‐43 uptake between the two groups of regenerated HCs (Figure 2K), it was found that the ability of FM1‐43 uptake in AAV‐AE group was stronger than that in AAV‐At group, indicating that the stereocilia function of AAV‐AE group was stronger and more mature in development. Therefore, we came to the conclusion that Espin overexpression promotes the development of stereocilia in regenerative HCs induced by Atoh1.

The maturation of HCs involves many complex developmental processes, such as the formation of hair bundles, synapses, and mechanotransduction channels,^25^ and thus the maturation of regenerated HCs is also related to the number of synapses that are formed. We injected the two viruses into the cochleae of P21 mice to determine their synaptic numbers by Ctbp2 staining (Figure 2L). The number of synapses in the AAV‐AE group was significantly greater than that in the AAV‐At group (Figure 2M), indicating that Espin overexpression promoted synapse formation in the regenerated inner HCs.

### 3.3 The effects of AAV‐Atoh1 injection on the stereocilia in endogenous and induced HCs

**FIGURE 3:**
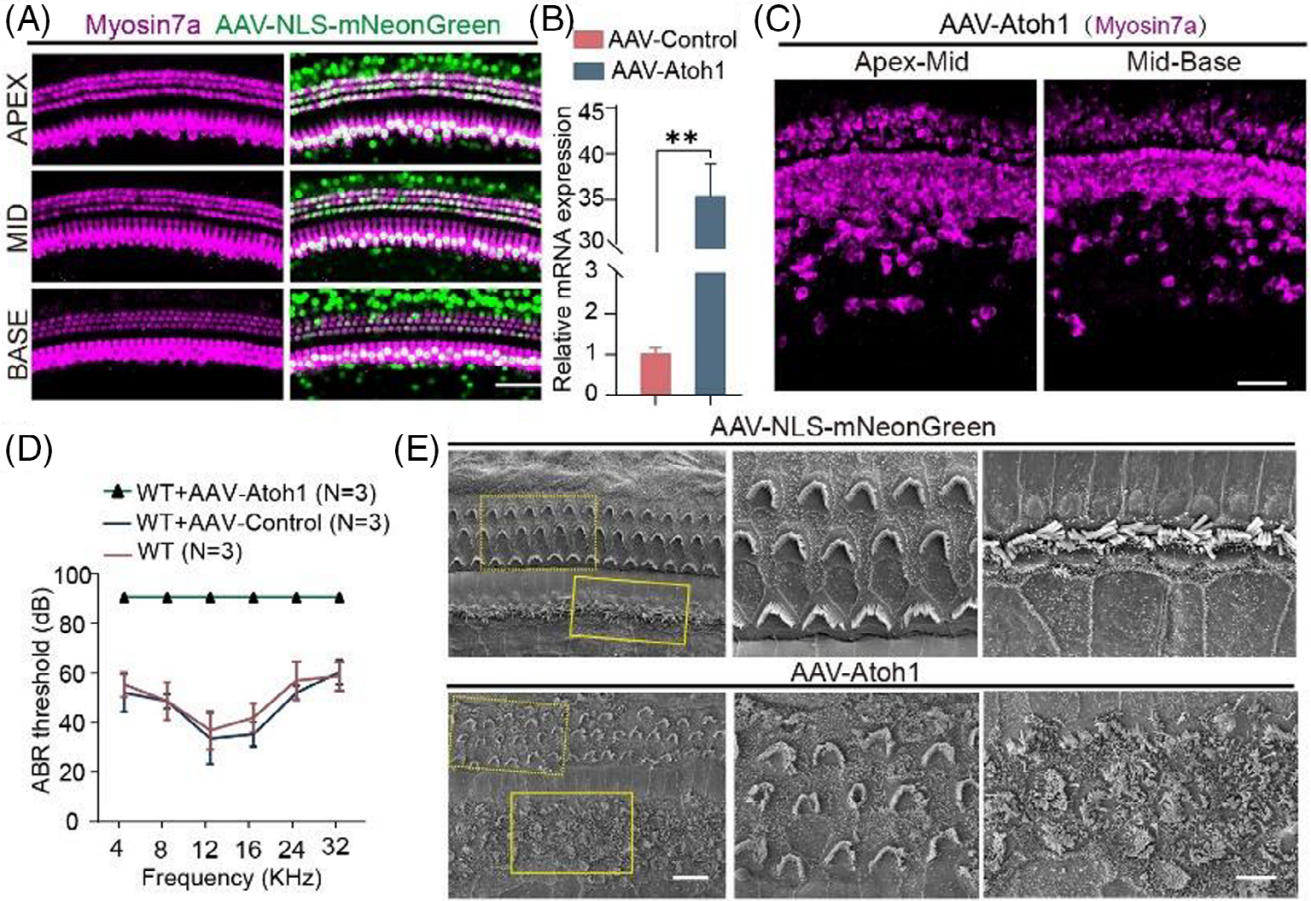
The effects of AAV‐Atoh1 injection on the hearing of mice. (A) Representative confocal images of Myosin7a (magenta) and AAV‐NLS‐mNeonGreen (green) without any gene overexpression. Scale bar, 50 μm. (B) Real‐time qPCR detected the expression of Atoh1 carried by AAV‐ie in vivo. (C) Confocal images of cochleae injected with AAV‐Atoh1. Magenta, myosin7a. Scale bar, 50 μm. (D) ABR audiometric data. (E) Representative SEM images of cochleae of P14 mice injected with AAV‐NLS‐mNeonGreen and AAV‐Atoh1. To the right are enlarged views of the yellow boxed regions. Scale bar; left, 10 μm, right, 5 μm. **p < 0.01.

In our previous experiments, we found that persistent overexpression of Atoh1 seems to have an effect on HCs in situ. To confirm this, we first needed to study the regeneration of HCs after AAV‐Atoh1 was injected into the cochlea through the round window membrane. Compared to the control group, Atoh1 induced the transdifferentiation of a large number of SCs into HCs in the inner HC region (Figure 3A,C), but due to the choice of viral vector Atoh1 also transfected large numbers of native HCs. Real‐time qPCR also verified that Atoh1 was overexpressed in vivo (Figure 3B). However, auditory brainstem response thresholds showed that mice injected with AAV‐Atoh1‐mNeonGreen became totally deaf, while mice injected with AAV‐mNeonGreen had normal hearing, indicating that HCs were likely to be damaged after Atoh1 overexpression in the cochlea (Figure 3D). Stereocilia are critical for the conversion of mechanical vibrations into action potentials by HCs, so we observed the morphology of the stereocilia. Through SEM we found that the stereocilia of the IHCs and OHCs in the experimental group were not arranged neatly, the opening direction was inconsistent, and some cilia were damaged (Figure 3E), and this explain why the mice became deaf after being injected with AAV‐Atoh1‐mNeonGreen.

### 3.4 Espin attenuates the damage to endogenous HCs induced by persistent Atoh1 overexpression

**FIGURE 4:**
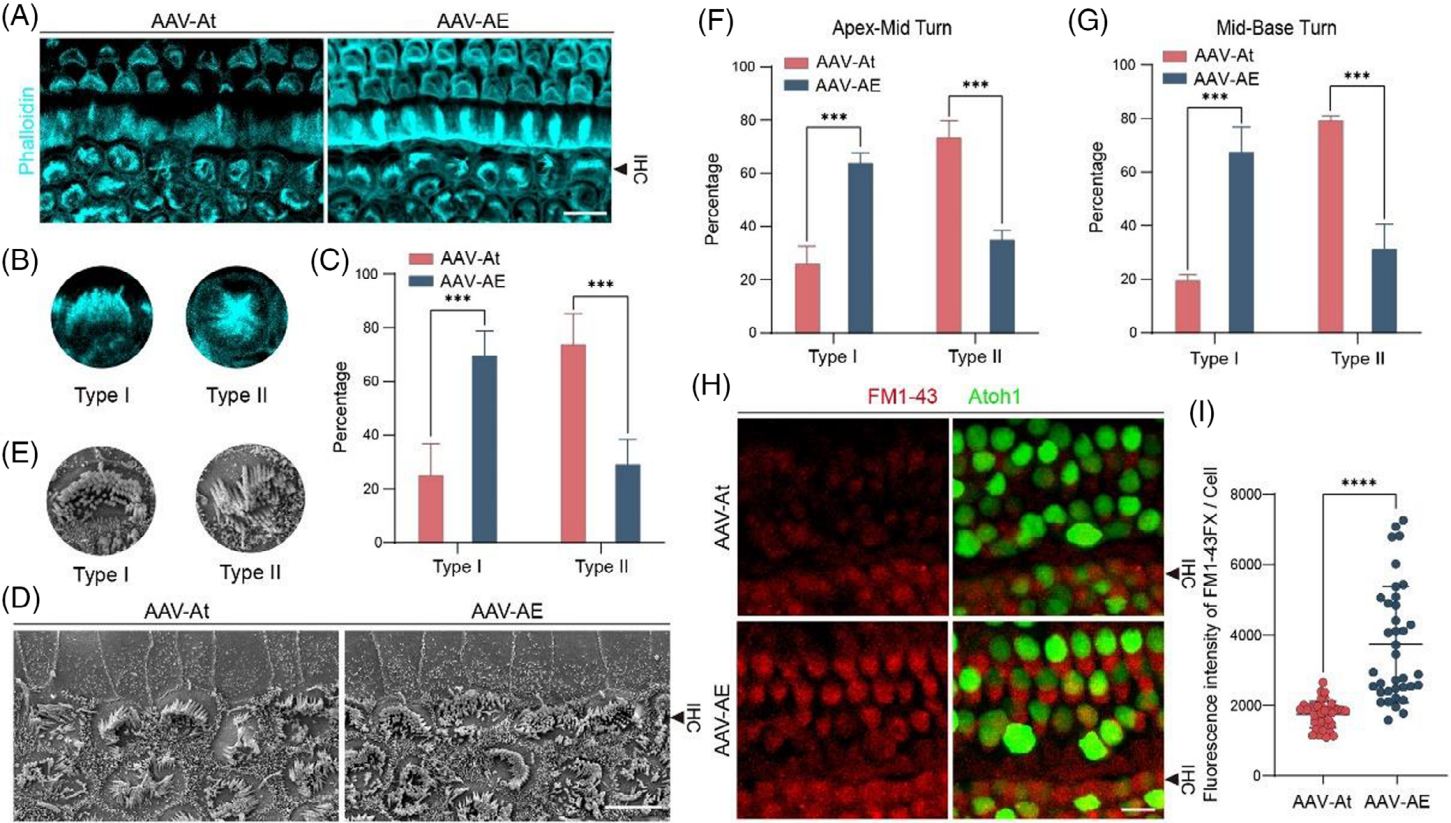
Stereocilia morphology and function of in situ hair cells after AAV injection. (A) Representative confocal images of stereocilia in endogenous hair cells. Scale bar, 10 μm. (B) Representative example diagram of in situ hair cell typing. (C) Stereocilia morphology was counted according to two types. (D) Representative SEM images of in situ hair cells. Scale bar, 5 μm. (E) Representative diagram of stereocilia typing. (F) Proportions of stereocilia morphology types in endogenous hair cells in the apex to middle of the cochlea. (G) Proportions of stereocilia morphology types in endogenous hair cells in the middle to base of the cochlea. (H) IHCs of P14 cochleae in two groups took up FM1‐43FX (red). Scale bar, 10 μm. (I) The fluorescence intensity of FM1‐43FX uptake analysis. ***p < 0.001; ****p < 0.0001.

Although Atoh1 promotes SC differentiation into HCs, endogenous HCs are unstable after overexpression of Atoh1, and the mature phenotype cannot be maintained according to the ciliary morphology (Figure 4A). The stereocilia morphology of IHCs in situ was also divided into two types, with type I being arc‐shaped and type II being ‘C’‐shaped or other shape (Figure 4B). The proportion of type I stereocilia in the AAV‐AE group was much higher than that in AAV‐At group, and the proportion of type II stereocilia was lower (Figure 4C). Type I stereocilia morphology was rather similar to that of wild‐type mouse IHCs, and in situ IHCs in the AAV‐AE group were less affected by Atoh1 overexpression, indicating that Espin could inhibit the effect of Atoh1 overexpression on endogenous IHCs.

Next, we performed statistical analysis on in situ stereocilia in SEM samples (Figure 4D). According to the degree of difference from wild‐type mouse IHCs, the stereocilia morphology of in situ IHCs was divided into two types according to the classification standard in the previous immunofluorescence picture (Figure 4E), and the proportion of type I stereocilia in the AAV‐AE group was much higher than that in AAV‐At group, and the proportion of type II stereocilia was lower (Figure 4F,G), which were consistent with the statistical results from the immunofluorescence experiments. These results suggest that Espin could inhibit the effect of persistent Atoh1 overexpression on HCs in situ.

To further understand the functional impairment in stereocilia, we added experiments of FM1‐43 uptake in cochlea AAV injected at P14 (Figure 4H). By comparing the fluorescence intensity of FM1‐43 uptake between the two groups of in situ HCs (Figure 4I), we found that the ability of FM1‐43 uptake in AAV‐AE group was stronger than that in AAV‐At group, indicating that the stereocilia function of in situ HCs in AAV‐AE group was better, thus proving that Espin overexpression indeed alleviates the stereocilia damage caused by Atoh1.

### 3.5 The expression of genes associated with stereocilia development

**FIGURE 5:**
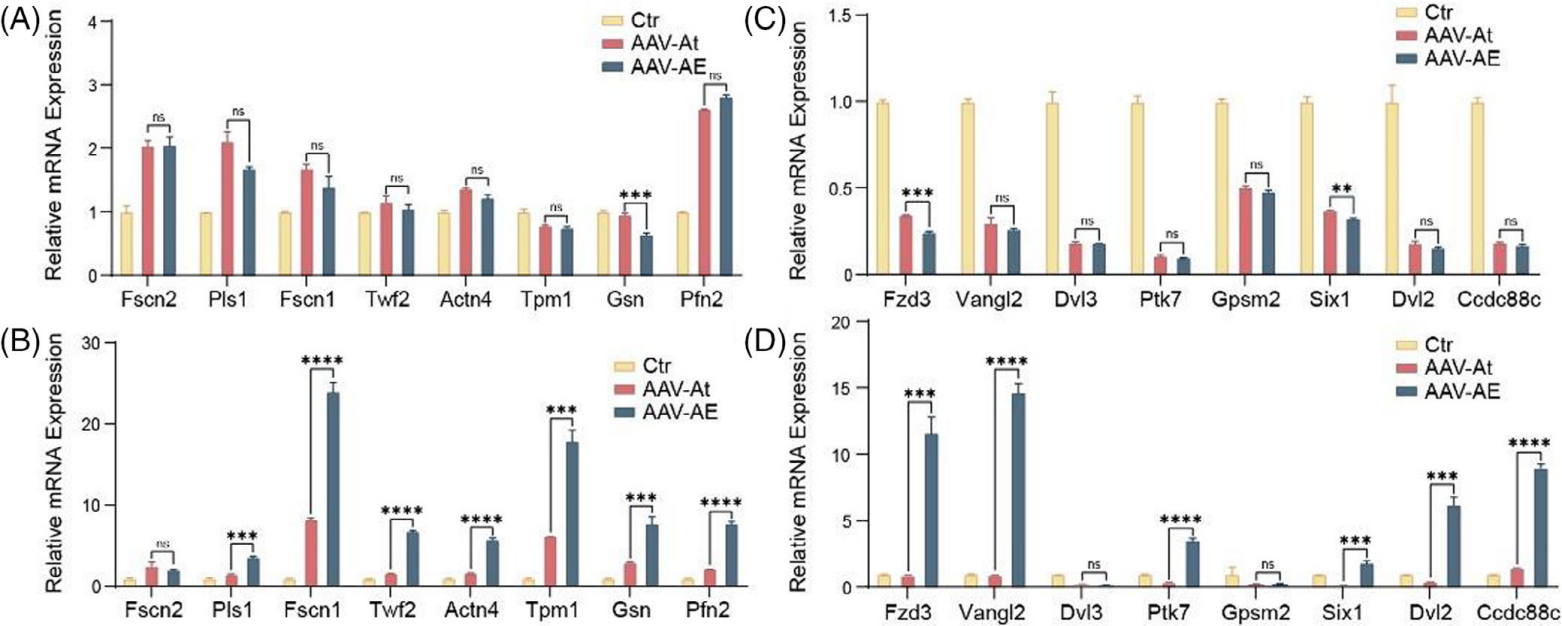
The expression of related genes at P7 and P14 mice. (A) The expression of genes associated with stereocilia‐building proteins at P7 was measured. (B) The genes were detected as (A), but samples were collected at P14. (C) The expression of genes associated with development of stereocilia regular morphology at P7 was measured. (D) The genes were detected as C, but samples were collected at P14. ns, no significance. **p < 0.01; ***p < 0.001; ****p < 0.0001.

Both the immunofluorescence and SEM results showed that Espin can promote the morphological development of stereocilia in neonatal HCs, and we detected the expression of relevant genes by real‐time qPCR to identify the mechanism behind this observation. We measured the expression of genes associated with stereocilia structural proteins at P7 and P14, including *Fscn2*, *Pls1*, *Fscn1*, *Twf2*, *Actn4*, *Tpm1*, *Gsn* and *Pfn2*. There was no significant difference in the expression of stereocilia structural protein‐related genes between the AAV‐AE and AAV‐At groups at P7 (Figure 5A), while the expression of most genes in the AAV‐AE group at P14 was higher than that in the AAV‐At group, and the expression of *Fscn1* was even more than 25 times that of the control group, indicating that stereocilia in the regenerated HCs at P14 developed rapidly (Figure 5B). In addition, we also examined the expression of genes associated with stereocilia plane polarity formation at P7 and P14, including *Fzd3*, *Vangl2*, *Dvl3*, *Ptk7*, *Gpsm2*, *Six1*, *Dvl2* and *Ccdc88c*. In HCs the stereocilia adopt a polarized structure in which rows of three‐dimensional cilia are arranged in a V‐shaped step pattern, and thus they are directionally sensitive to mechanical stimuli. At P7, there was no significant difference in the expression of relevant genes in the AAV‐AE and AAV‐At groups (Figure 5C), possibly because the stereocilia were still in a newly developed state at that time and the polarity formation at the cellular level had not yet begun. However, the expression of related genes in the AAV‐AE group at P14 was significantly higher than that in the AAV‐At group (Figure 5D), suggesting that the polarity of regenerated IHCs in the AAV‐AE group at P14 was closer to that of in situ inner HCs. These results indicate that overexpression of Espin promotes the expression of polarity‐related genes in HCs, resulting in more regular stereocilia morphology.

## 4 DISCUSSION

In mammals, the loss of HCs leads to permanent sensorineural hearing loss because HCs cannot spontaneously regenerate in the mature cochlea. In contrast, HCs in non‐mammalian vertebrates can regenerate after damage, rebuilding related hearing functions. Therefore, we have considered whether hearing might be restored in mammals through a similar pattern. Studies have shown that influencing transcription factors and signalling pathways can induce SCs to transdifferentiate into HCs in mammals, which brings great hope for the treatment of hearing loss.^25^, ^26^ Gene therapy has proven to be an efficacious tool for clinical applications to correct gene defects and introduce beneficial gene expression into a variety of tissues, and recently gene therapy has made great strides in the treatment of genetic inner ear diseases.^27^, ^28^ Thus, the prospects of regenerating HCs through gene therapy to restore hearing is very promising.

We observed no aggregated actin fibres in HEK293T cells during culture, but cells with Espin overexpression produced clearly visible actin bundles, which verified that Espin promotes the formation of actin bundles.^29^ In organoid and explant culture, Atoh1 overexpression promoted the differentiation of SCs into HCs, but the stereocilia of the neonatal HCs were short or even nonexistent, which was consistent with the results of previous studies.^11^, ^21^ Correspondingly, co‐overexpression of Espin and Atoh1 promoted the growth of stereocilia‐like structures in regenerated HCs. During organoid culture, there may be no space for cilia to grow due to the tight connections between cells. In explant culture, HCs induced only by Atoh1 rarely grew cilia, and even though stereocilia‐like structures grew in the AAV‐AE group, the cilia were extremely messy and non‐directional. We speculated that the formation of stereocilia may require regulation by multiple genes, and in the in vitro environment Atoh1 alone could not successfully stimulate the expression of stereocilia‐related genes. However, Espin could promote actin fibre polymerization, thus forming stereocilia‐like structures in coordination with related genes. This suggests that the genetic programme for stereocilia development in regenerated HCs is partially active, but in vitro stimulation with Espin overexpression is required.

Similar to the results of other studies,^23^, ^30^ persistent overexpression of Atoh1 in native HCs could cause loss of the stepped structure and a shortening of the stereocilia. Therefore, the persistent overexpression of Atoh1 during the experiment would also cause damage to the stereocilia of regenerative HCs, which is one of the reasons why only Atoh1 overexpression induces the disorder of stereocilia in regenerated HCs. Espin is an actin binding protein and plays a key role in stereocilia development in HCs,^17^, ^21^, ^29^ and we show here that upregulation of Espin in HCs can inhibit damage to stereocilia caused by continuous expression of Atoh1. This may be because Espin overexpression maintains the expression of genes related to stereocilia formation such that Atoh1 overexpression can no longer disrupt this process.

The length of stereocilia in regenerated HCs in vivo is significantly different from that of explants and is closer to that of HCs in situ. In addition, we found that in vivo Espin overexpression did not significantly increase the length of stereocilia in neonatal HCs. However, a previous study found that the length of stereocilia increased and became thicker at P60 after Espin overexpression,^20^ which was inconsistent with our results. We increased the expression of Espin in regenerated HCs and in situ HCs with persistent overexpression of Atoh1, rather than HCs unaffected by Atoh1, where the expression of Atoh1 causes changes in HCs, so the stereocilia changes caused by Espin overexpression are inconsistent with the previous research. These results all indicate that there is a large difference in the expression of relevant genes in vitro and in vivo. Through real‐time qPCR, it could be seen that at P7 and P14 the expression of most genes associated with cilia‐constituent proteins in the AAV‐At and AAV‐AE groups was not very different, which explains why there was no significant difference in stereocilia length between the two groups in vivo. In our present study, stereocilia could occur spontaneously when regenerated HCs were induced only by Atoh1 overexpression in vivo, and Espin overexpression had no significant effect on the length of stereocilia but promoted the regularization of the stereocilia morphology in regenerated HCs. It has been suggested that the centrifugal migration of the HC primary cilium, the kinocilium, and its associated basal body occurs towards the lateral pole of the cell.^31^ We divided the morphology of stereocilia of regenerated HCs in SEM into four types, and the change from type IV to type I was consistent with the degree of development maturity of stereocilia in situ mentioned in the article, and the proportion of type I in the AAV‐AE group increased significantly. Based on our real‐time qPCR results, we speculate that the expression of planar polarity‐related genes increases after Espin overexpression, thereby promoting the stereocilia development of neonatal HCs. One of the characteristics of HCs exhibiting planar polarity is that the rows of stereocilia are arranged in a V‐shaped step pattern and thus are directionally sensitive to mechanical stimulation.^32^, ^33^ Therefore, regenerated HCs with Espin overexpression might also exhibit better physiological functions when the planar polarity of neonatal HCs is improved.

AAV‐mediated Atoh1 overexpression also has a problem because AAV is stable within cells and more SCs are found to transdifferentiate into HCs over time. The proliferation rate of SCs is unknown, and thus it is not known if the loss of a large number of SCs due to differentiation into HCs might have other negative effects. Ideally, we only want Atoh1 to be overexpressed for a short period of time so as not to have a significant effect on other cells.

In conclusion, we found that Espin overexpression could promote the stereocilia development of Atoh1‐induced HCs and could attenuate the damage caused by persistent overexpression of Atoh1 to HCs in situ. Although the development of neonatal HCs cannot yet achieve the morphology of in situ HCs, this represents an important step forward in the production of fully functional HCs in the mammalian cochlea.

## AUTHOR CONTRIBUTIONS

Peina Wu conceived the study. Renjie Chai, Jieyu Qi and Yong Cui conceived and supervised the project. Xuechun Yang conducted most of the experiments and acquired data with the help from Liyan Zhang and Fangzhi Tan. Liyan Zhang, Hongming Huang and Chunlai Xu contributed to data interpretation and analysis. Peina Wu wrote the manuscript. Jieyu Qi, Renjie Chai and Yong Cui reviewed the manuscript. All authors approved the final manuscript.

## FUNDING INFORMATION

This work was supported by grants from the National Key R&D Program of China (No. 2021YFA1101300, 2021YFA1101800, 2020YFA0112503, 2020YFA0113600), the National Natural Science Foundation of China (Nos. 82030029, 81970882, 82000984, 92149304), the China National Postdoctoral Program for Innovative Talents (BX20200082), the China Postdoctoral Science Foundation (2020M681468), the Jiangsu Postdoctoral Research Funding Program (2021K156B), the Science and Technology Department of Sichuan Province (No. 2021YFS0371), the Shenzhen Fundamental Research Program (JCYJ20190814093401920, JCYJ20210324125608022), the Open Research Fund of the State Key Laboratory of Genetic Engineering, Fudan University (No. SKLGE‐2104), the 2022 Open Project Fund of Guangdong Academy of Medical Scienses (YKY‐KF202201), the Initial funding of the National Natural Science Foundation of China (8217040403) and the Guangdong Basic and Applied Basic Research Foundation (2021A1515220179).

## CONFLICT OF INTEREST STATEMENT

The authors declare no competing interest.

## Data Availability

All data associated with this study are presented in the paper.
